# Differences in SARS-CoV-2-Specific Antibody Responses After the First, Second, and Third Doses of BNT162b2 in Naïve and Previously Infected Individuals: A 1-Year Observational Study in Healthcare Professionals

**DOI:** 10.3389/fimmu.2022.876533

**Published:** 2022-05-27

**Authors:** Manca Ogrič, Polona Žigon, Eva Podovšovnik, Katja Lakota, Snezna Sodin-Semrl, Žiga Rotar, Saša Čučnik

**Affiliations:** ^1^ Department of Rheumatology, University Medical Centre Ljubljana, Ljubljana, Slovenia; ^2^ University of Primorska, Faculty of Mathematics, Natural Sciences and Information Technologies, Koper, Slovenia; ^3^ Valdoltra Orthopaedic Hospital, Ankaran, Slovenia; ^4^ Faculty of Medicine, University of Ljubljana, Ljubljana, Slovenia; ^5^ Faculty of Pharmacy, University of Ljubljana, Ljubljana, Slovenia

**Keywords:** COVID-19, SARS-CoV-2, BNT162b2 vaccine, anti-S antibody dynamics, healthcare professionals, humoral immune response

## Abstract

**Background:**

Safe and effective vaccines against COVID-19 are critical for preventing the spread of SARS-CoV-2, but little is known about the humoral immune response more than 9 months after vaccination. We aimed to assess the humoral immune response after the first, second, and third (booster) doses of BNT162b2 vaccine in SARS-CoV-2 naïve and previously infected healthcare professionals (HCP) and the humoral immune response after infection in vaccinated HCP.

**Methods:**

We measured anti-spike (anti-S) and anti-nucleocapsid antibodies at different time points up to 12 months in the sera of 300 HCP who had received two or three doses of BNT162b2 vaccine. Mixed-model analyses were used to assess anti-S antibody dynamics and to determine their predictors (age, sex, BMI, and previous infection).

**Results:**

Naïve individuals had statistically lower anti-S antibody concentrations after the first dose (median 253 BAU/ml) than previously infected individuals (median 3648 BAU/ml). After the second dose, anti-S antibody concentrations increased in naïve individuals (median 3216 BAU/ml), whereas the second dose did not significantly increase concentrations in previously infected individuals (median 4503 BAU/ml). The third dose resulted in an additional increase in concentrations (median 4844 BAU/ml in naïve and median 5845 BAU/ml in previously infected individuals). Anti-S antibody concentrations steadily decreased after the second dose and after the third dose in naïve and previously infected individuals. In addition, we found that age had an effect on the humoral immune response. Younger individuals had higher anti-S antibody concentrations after the first and second doses. After infection with the new variant Omicron, a further increase in anti-S antibody concentrations to a median value of 4794 BAU/ml was observed in three times vaccinated HCP whose anti-S antibody concentrations were relatively high before infection (median 2141 BAU/ml). Our study also showed that individuals with systemic adverse events achieved higher anti-S antibody concentrations.

**Conclusion:**

In this study, significant differences in humoral immune responses to BNT162b2 vaccine were observed between naïve and previously infected individuals, with age playing an important role, suggesting that a modified vaccination schedule should be practiced in previously infected individuals. In addition, we showed that the high anti-S antibodies were not protective against new variants of SARS-CoV-2.

## 1 Introduction

The pandemic of coronavirus disease 2019 (COVID-19) caused by severe acute respiratory syndrome coronavirus 2 (SARS-CoV-2) has greatly affected the normal life of people and the functioning of society (1). One of the key factors to prevent the spread of infection in the population is the use of safe and effective vaccines. Vaccination protects individuals and thus reduces the risk of clinically significant consequences in the event of infection. An additional issue in protecting vaccinated and previously infected individuals is the new variants of the virus, including Omicron, that are emerging in the population (2, 3).

The efficacy of different vaccines has been reported in clinical trials to be 50-95% (4–7). The efficacy of two doses of the vaccine BNT162b2 (Comirnaty, Pfizer and BioNTech), a single-stranded mRNA carrying the spike (S) protein transcript, located in lipid nanoparticles, is 95% (4), while protection against reinfection in previously infected individuals is estimated at 89% (7). The specific antibodies against SARS-CoV-2 produced during infection are antibodies against S and nucleocapsid protein (N) (anti-S and anti-N antibodies), whereas only anti-S antibodies are produced after vaccination with BNT162b2. The duration of protection that an individual develops after vaccination or after overcoming infection is still largely unclear. However, we do know that the primary humoral immune response declines over time (8–10). In addition, information about concentrations of anti-S antibodies sufficient to prevent infection is not known but would be clinically relevant, especially for the new Omicron variant.

Currently, there are limited data on the dynamics and concentrations of anti-S antibodies over a longer period after vaccination. A clinical study investigating antibody responses after initial and booster vaccination with BNT162b2 has shown strong anti-S IgG antibody responses with concentrations exceeding those in COVID-19 convalescent plasma and a decrease in antibody concentrations 85 days after the first dose (11). Longer studies, as presented by Collier et al. showed data in which decreasing vaccine immunity was observed 8 months after vaccination, not only with BNT162b2 but also with other vaccines used (12).

Responses to vaccination also vary widely due to differences between individuals. In some studies, a negative correlations between anti-S antibody concentrations and age were observed (13–16), while one study showed lower magnitude of memory B-cell responses with increasing age (17). Limited information is available on association of adverse events and anti-S antibody concentration. Specifically, Goel et al. found no significant correlation between anti-S antibody concentration and the severity of adverse events (17), while Naaber et al. reported that adverse events correlated positively with anti-S antibody concentration (13).

In our study, we primarily investigated the humoral immune response to the first, second, and third (booster) doses of BNT162b2 vaccine by measuring anti-S antibody concentrations in healthcare professionals (HCP) who were not previously infected with SARS-CoV-2 virus (so-called naïve individuals) and in previously infected individuals. We also examined the 12-month dynamics of anti-S antibody concentrations after the second dose and the 3-month dynamics after the third (booster) dose, as well as the concentrations of anti-S antibodies after infections with SARS-CoV-2 in vaccinated HCP. In addition, we performed a mixed-model analyses to determine the predictors (age, sex, BMI and previous infection) of anti-S antibody concentrations and dynamics. Finally, we compared anti-S antibody concentrations among the group of HCP with systemic, local, and no adverse events after the second dose.

## 2 Material and Methods

### 2.1 Sample and Data Collection

HCP, employees of the Division of Internal Medicine University Medical Centre Ljubljana, who had received at least two or three doses of the BNT162b2 mRNA vaccine were included in the study. Participants provided detailed information on their health status (presence of chronic diseases), demographics, lifestyle (age, sex, BMI), occurrence of adverse events after vaccination, and SARS-CoV-2 infection before and during vaccination *via* questionnaires.

**Figure 1:**
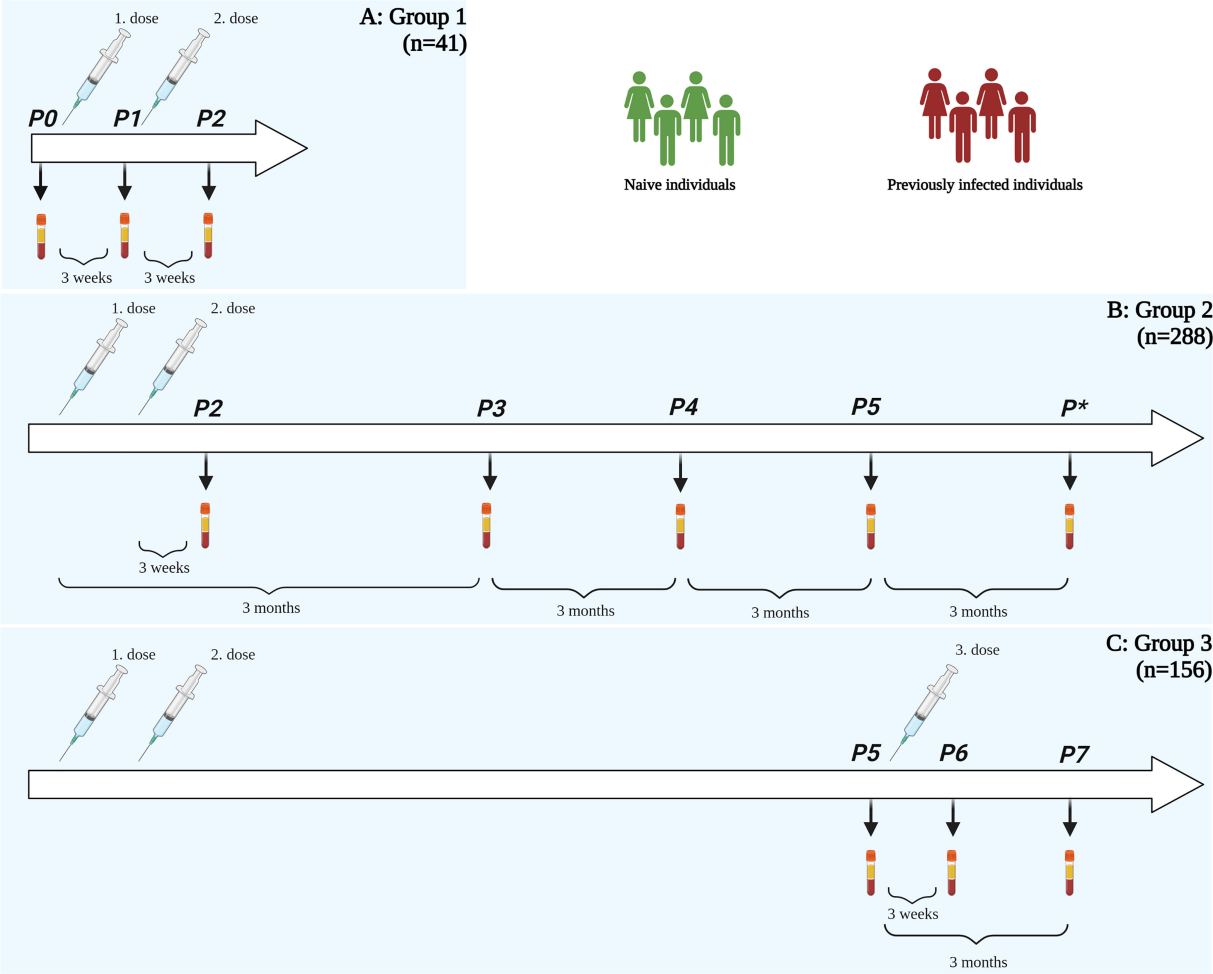
Summary of the study. **(A)** Group 1: participants with samples in P0, P1, and P2 (n = 41) **(B)** Group 2: participants with samples in P2, P3, P4, and P5 (at least two samples, n = 288) and **(C)** Group 3: participants with samples in P5, P6 and P7 (n = 156). Legend: P0 – time point before vaccination, P1 – time point three weeks after first dose, P2 – time point three weeks after second dose, P3 – time point three months after vaccination, P4 – time point six months after vaccination, P5 – time point nine months after vaccination, P* – time point twelve months after vaccination, P6 – time point three weeks after third dose, P7 – time point three months after third dose.

Serum samples from HCP were collected longitudinally from December 2020 to December 2021 at different time points: before the first dose (P0), three weeks after the first dose (P1), approximately three weeks (range 2-5 weeks) after the second dose (P2), three (P3), six (P4), nine (P5) and twelve (P*) months after the first dose; and three weeks (P6) and three months (P7) after the third (booster) dose (**Figure 1**). Additional samples were collected from January to February 2022 only from HCP infected with SARS-CoV-2 after the last time point (P6 or P7).

The study was approved by the Slovenian National Medical Ethics Committee (#0120-422/2020/6). Participants signed an informed consent form before recruitment to the study. The study was conducted in accordance with the Helsinki Declaration.

### 2.2 Antibody Testing

Anti-S antibodies to the S1 subunit and anti-N antibodies were measured by enzyme linked immunosorbent assay (anti-SARS-CoV-2 QuantiVac ELISA (IgG) for detection of anti-S antibodies and anti-SARS-CoV-2 NCP ELISA (IgG) for detection of anti-N antibodies, both from Euroimmun, Germany). Concentrations of anti-S antibodies were used to evaluate the efficacy of vaccination, while the presence of anti-N antibodies indicated symptomatic, PCR-positive individuals and asymptomatic COVID-19 infections. ELISAs were performed according to the manufacturer`s instructions.

The manufacturer has calibrated the units of anti-S antibody concentration to the first World Health Organization (WHO) standard (NIBSC code: 20/136) and recommends reporting results in binding antibody units per milliliter (BAU/ml), with values <25.6 BAU/ml reported as negative. In the case of anti-N antibodies, the ratio <0.8 is considered a negative result.

### 2.3 Statistics

Statistical significances were determined by nonparametric statistical tests (for independent samples - Mann-Whitney U test and Kruskal-Wallis test with Dunn’s multiple comparison test, for dependent samples – Wilcoxon signed-rank test). Mixed models were used to assess the dynamics of anti-S antibodies (dependent variable) and relate these changes to age groups (<45 years and >45 years), sex, BMI groups (<25 and >25), and previous infections (fixed covariates). GraphPad Prism 8 and SPSS IBM 25 were used for statistical analysis.

## 3 Results

The study included 300 HCP [female 239 (80%), male 61 (20%), median age 43 (IQR 35-53)]. 292 HCP provided information on chronic diseases and medications. 69/292 (24%) HCP had one or more chronic diseases (arterial hypertension, cardiovascular disease, diabetes, chronic kidney disease, chronic rheumatic disease, inflammatory bowel disease, Hashimoto`s disease, asthma…) and five of them reported receiving immunosuppressive drugs.

The participants were divided into three study groups according to the time points they had their sera collected (**Figure 1**):

- group 1: 41 participants, with serum samples collected before and following the first and second doses (time points P0, P1 and P2),
- group 2: 288 participants who sent at least two of the four samples after the second dose (time points P2, P3, P4 and P5). Within this group, 22 individuals did not receive a third dose and had their serum collected also after 12 months (P*).
- group 3: 156 participants who sent their serum samples after receiving the third (booster) dose (time point P6) and 69 participants who sent their serum samples 3 months after the third (booster) dose (time point P7).

During our study, eleven HCP were infected between the first and second dose, four between three and nine months, four between nine and twelve months after vaccination and five after the third dose. Between January and February 2022, when the Omicron variant was the predominant one, an additional 27 HCP were infected. The results of their anti-S antibody concentrations are presented separately.

### 3.1 Humoral Immune Response After Vaccination

#### 3.1.1 Humoral Immune Response After the First Dose

The humoral immune response to the first dose was analyzed in the group, which included 41 HCP, 29 were naïve and 12 were previously infected individuals (8 with known infection confirmed by a positive PCR test before vaccination and 4 asymptomatic individuals with positive anti-S and/or anti-N antibodies before vaccination). Anti-N antibody levels were also tested in these samples. In the previously infected individuals, the median anti-N antibody level was 1.67 (ratio). There was no correlation between anti-N and anti-S antibody concentrations in these 12 participants. Interestingly, we found a negative correlation -0.652 (p=0.02) between anti-S antibody concentrations in P0 and the ratio of anti-S antibody concentrations between P1/P0, suggesting that those with lower anti-S antibody concentrations before vaccination had a higher increase in anti-S antibody concentration after the first dose than those with higher anti-S antibody concentrations before vaccination.

**Figure 2:**
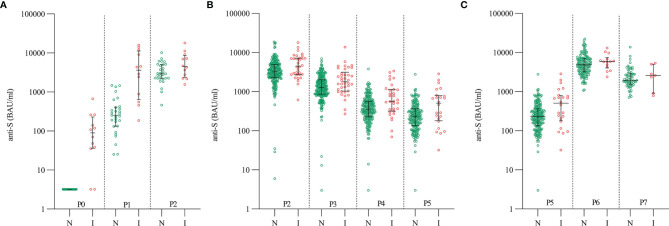
Dynamics of anti-S antibody concentrations after vaccination with BNT162b2 in naïve (green) and previously infected (red) individuals: **(A)** Group 1: before, after the first and second doses (P0, P1 and P2), **(B)** Group 2: after the second dose (P2, P3, P4 and P5) and **(C)** Group 3: after the third dose (P5, P6 and P7). N – naïve individuals (green color), I – previously infected individuals (red color), P0 – time point before vaccination, P1 – time point three weeks after first dose, P2 – time point three weeks after second dose, P3 – time point three months after vaccination, P4 – time point six months after vaccination, P5 – time point nine months after vaccination, P6 – time point three weeks after third dose, P7 – time point three months after third dose.

In naïve anti-S antibody concentration before the first dose was median 3.2 BAU/ml and after vaccination 253 BAU/ml, whereas in previously infected individuals it was 90 BAU/ml before and 3648 BAU/ml after the first dose. Thus, individuals with previous infection had significantly higher concentrations of anti-S antibodies before vaccination (p<0.001) and after the first dose (p<0.001) (**Table 1** and **Figure 2A**). Anti-S antibody concentrations increased after the first dose in both naïve (median 79-fold change) and in previously infected individuals (median 52-fold change).

**Table 1:** Comparison of anti-S antibody concentrations between naïve and previously infected individuals in all time-points.

		Naïve individuals	Previously infected individuals	Mann Whitney; p
**P0**	**anti-S (BAU/ml), median (IQR)**	3.2 (3.2-3.2)	90 (36-230)	*<0.001*
	**n**	29	12	
**P1**	**anti-S (BAU/ml), median (IQR)**	253 (133-411)	3648 (638-11278)	*<0.001*
	**n**	29	12	
**P2**	**anti-S (BAU/ml), median (IQR)**	3216 (2278-4925)	4503 (2731-7201)	*0.008*
	**n**	227	32	
**P3**	**anti-S (BAU/ml), median (IQR)**	1293 (858-2006)	1784 (1031-3146)	*0.008*
	**n**	240	38	
**P4**	**anti-S (BAU/ml), median (IQR)**	355 (228-569)	563 (318-1133)	*0.001*
	**n**	213	31	
**P5**	**anti-S (BAU/ml), median (IQR)**	232 (134-356)	507 (181-801)	*0.008*
	**n**	198	24	
**P6**	**anti-S (BAU/ml), median (IQR)**	4844 (3215-6984)	5845 (4039-7495)	*0.32*
	**n**	142	14	
**P7**	**anti-S (BAU/ml), median (IQR)**	1951 (1545-2967)	2586 (915-5053)	*0.60*
	**n**	62	7	
P*	**anti-S (BAU/ml), median (IQR)**	83 (51-134)	229 (101-417)	*0.09*
	**n**	17	5	

P0 – time point before vaccination, P1 – time point three weeks after first dose, P2 – time point three weeks after second dose, P3 – time point three months after vaccination, P4 – time point six months after vaccination, P5 – time point nine months after vaccination, P6 – time point three weeks after third dose, P7 – time point three months after third dose, P* – time point twelve months after vaccination.

#### 3.1.2 Humoral Immune Response After the Second Dose

Humoral immune response after the second dose was assessed in 288 HCP, 41 HCP had been previously infected (25 with positive PCR test and 16 with positive anti-N antibodies, with a median anti-N antibody level of 2.23).

Three weeks after the second dose, median concentration of anti-S antibodies was 3216 BAU/ml in naïve individuals and 4503 BAU/ml in previously infected individuals (**Table 1** and **Figure 2B**). However, after the second dose a significant increase (median 13-fold change) was only observed in naïve individuals. In previously infected individuals, an increase after the second dose was not statistically significant. Importantly, antibody concentrations measured in previously infected individuals after the first dose were comparable to those measured in naïve individuals after the second dose (p = 0.83), pointing toward same humoral immune response in second encounter with viral proteins. In naïve individuals, median concentrations of anti-S antibodies at different time points after the second dose showed a decrease and were 1293 BAU/ml at three months, 355 BAU/ml at six months, and 232 BAU/ml at nine months after vaccination. In previously infected individuals, median concentration of anti-S antibodies were 1784 BAU/ml, 563 BAU/ml, and 507 BAU/ml at three, six, and nine months after vaccination, respectively. At all-time points, previously infected individuals had higher anti-S antibody concentrations than naïve individuals (**Table 1** and **Figure 2B**). One naïve participant did not develop anti-S antibodies after vaccination (value < 25.6 BAU/ml), and in two naïve participants anti-S antibody concentrations fell below 25.6 BAU/ml three months after vaccination. It is important to note that, two of these three participants have been treated with immunosuppressive therapy due to chronic diseases, while the third one was not receiving any immunosuppressive drugs.

In addition, 22 individuals who had not yet been vaccinated with the third dose had their sera withdrawn 12 months after they received the first dose. Five of them were previously infected individuals and tthe median concentration of anti-S antibodies was 229 BAU/ml, while the naïve individuals had a median concentration of anti-S antibodies of 83 BAU/ml 12 months after vaccination. Due to the small numbers, no statistical difference between the groups could be detected (**Table 1**).

#### 3.1.3 Humoral Immune Response After the Third (Booster) Dose

Humoral immune response after the third (booster) dose was assessed in 156 HCP. Among them, 14 HCP had been previously infected (6 with positive PCR test and 8 with positive anti-N antibodies, with a median anti-N antibody level of 1.65).

Three weeks after the third dose, the median concentrations of anti-S were 4844 BAU/ml in naïve individuals and 5845 BAU/ml in previously infected individuals, both groups having an increase in anti-S concentration compared to levels before receiving the third dose. There was a slightly higher median fold change in naïve individuals, that is 22-fold increase in concentrations after the third dose in comparison to concentrations before receiving the third dose, and 17-fold increase in concentrations in previously infected individuals. Three months after the third dose, 69 samples were analyzed, and median concentration of anti-S antibodies was 1951 BAU/ml in naïve individuals and 2586 BAU/ml in previously infected individuals (**Table 1** and **Figure 2C**).

#### 3.1.4 Comparison Between Humoral Immune Responses After the Second and After the Third Dose

In addition, we found that the concentrations of anti-S antibodies were higher after the third dose than after the second dose. This difference was statistically significant in naïve (p<0.001) and previously infected individuals (p=0.033).

Additionally, we found a negative correlation between the ratio of the anti-S antibody concentrations after the third dose and after the second dose (P6/P2 ratio) and the anti-S antibody concentrations after the second dose (r=-0.501, p<0.001), showing that those individuals who had lower concentrations after the second dose had later higher concentration after the third dose. In this part both groups (naïve and previously infected individuals) were combined and analyzed together, as there was no significant difference in the P6/P2 ratio between the groups (p=0.31).

#### 3.1.5 The Influence of Age, Sex, BMI, and Previous Infection on Anti-S Antibody Concentrations and Their Dynamics Using Mixed-Model Analysis

The results of the mixed-model analyses with multiple variables (age groups, sex, BMI groups, and previous infection) are presented in **Supplementary Table 1** for groups 1, 2, and 3, first for the entire group and then for the naïve individuals only.

In group 1, we found that previous infection influenced the anti-S antibody concentrations reached after the first and second doses. The results showed that previously infected individuals had higher anti-S antibody concentrations after the first and second dose than naïve individuals. In the group of naïve individuals only, an influence of age was additionally found. That is, individuals in the naïve group who were younger than 45 years had higher anti-S antibody concentrations after the first dose and after the second dose than those who were older than 45 years. Over time, anti-S antibody concentrations in this group increased after the first and second dose until time point P2.

In group 2, anti-S antibody concentrations were related to previous infection and age: individuals younger than 45 years and those with previous infection had higher anti-S antibody concentrations after the second dose. In naïve individuals, age again influenced anti-S antibody concentrations after the second dose. Anti-S antibody concentrations decreased in this group from time point P2 to P5.

Interestingly, in group 3, there was no longer an influence of previous infection, but we found that age and BMI influenced anti-S antibody concentrations after the third dose. Importantly, in this case, individuals older than 45 years and with higher BMI had higher concentrations of anti-S antibodies. In the naïve group, no influence of age was observed. Again, anti-S antibody concentrations decreased after the third dose until time point P7.

### 3.2 Outcomes of HCP Who Became Infected During the Observation Period

Eleven HCP who were infected with SARS-CoV-2 between the first and second dose had median anti-S antibody concentration after the second dose of 4195 BAU/ml (P2), 1975 BAU/ml (P3), 705 BAU/ml (P4) and 502 BAU/ml (P5) and after the third dose 5658 BAU/ml. Median concentrations after the second dose in these individuals were comparable to previously infected individuals (with infection before vaccination) (no significant differences in P2, P3, P4 and P5) and were higher than in naïve individuals (statistical differences in P3 (p=0.04) and P5 (p=0.03), P2 and P4 – not significant). After the third dose, no statistically significant difference in anti-S antibody concentrations between these HCP and naïve or previously infected individuals was observed.

Four HCP were infected after the second dose (three between 3 and 6 months, and one between 6 and 9 months). Two of them had positive PCR, while two of them had asymptomatic disease. In all these individuals, anti-S and anti-N antibodies increased after their infection. Their median anti-S antibody concentration after vaccination and before infection was 430 BAU/ml which increased to median concentration 2490 BAU/ml after infection.

In addition, four HCP were infected later, between 9 and 12 months after complete vaccination, all four with positive PCR. The median anti-S antibody concentration before infection was 90 BAU/ml at nine months after vaccination and increased to 4942 BAU/ml after infection. The increase after infection was significant and similar to concentrations after the third dose in naïve individuals.

Furthermore, five HCP were infected 3 weeks after the third dose, when their anti-S antibody levels were high (median 3475 BAU/ml).

### 3.3 Infections in HCP During the Period of High Prevalence of Omicron Variant

Four months after the third dose the Omicron variant became predominant. 27 HCP were infected during the following two-month period. Their median concentration of anti-S antibodies before infection was 2141 BAU/ml (IQR 1536-3194, samples were taken 22.5 days (median) before infection, IQR 17-41 days). After infection, anti-S antibody concentrations increased to a median concentration of 4794 BAU/ml (IQR 3414-5392) (p<0.001).

### 3.4 Adverse Events and Anti-S Antibody Concentrations After the Second Dose

**Figure 3:**
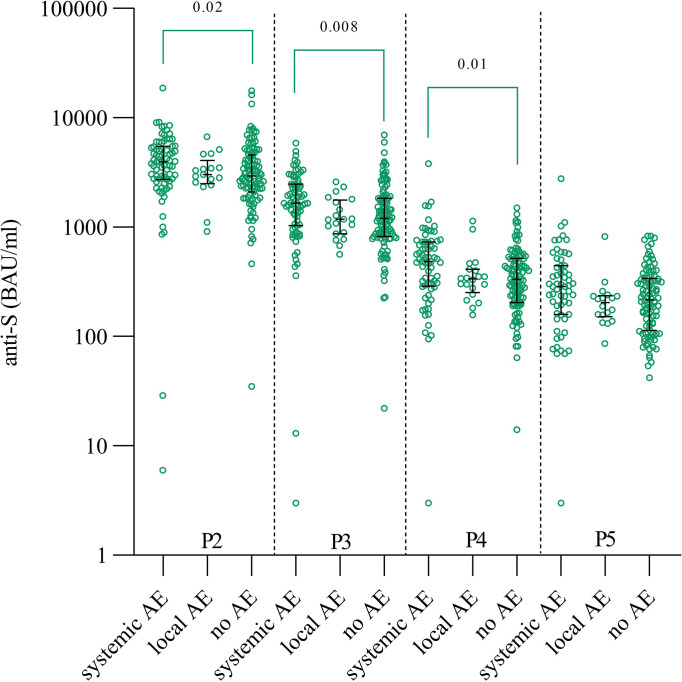
Anti-S antibody concentrations in individuals with systemic, local and no adverse events after the second dose. Statistical differences were determined by Kruskal-Wallis test together with Dunn’s multi-comparison test (significances on the graphs). AE – adverse events, P2 – time point three weeks after second dose, P3 – time point three months after vaccination, P4 – time point six months after vaccination, P5 – time point nine months after vaccination.

249/300 (83%) participants properly answered the questionnaire on the occurrence of adverse events. Of these, 135/249 (54%) subjects had no adverse events after vaccination, while 114/249 (46%) subjects reported various adverse events such as headache, fatigue, chills, fever, muscle pain, and redness at the injection site. Subjects with adverse events were divided into two groups according to the occurrence of local or systemic adverse events and compared with subjects reporting no adverse events. Since we had shown that anti-S antibody concentrations differed between patients and naïve subjects, we also considered the latter. We found that naïve individuals with systemic adverse events had higher concentrations of anti-S antibodies at all four time points (P2, P3, P4 and P5) after vaccination (median 3955 BAU/ml, 1651 BAU/ml and 487 BAU/ml and 285 BAU/ml, respectively) compared with those with local adverse events (median 3034 BAU/ml, 1179 BAU/ml and 335 BAU/ml and 205 BAU/ml, respectively) or no adverse events (median 2947 BAU/ml, 1205 BAU/ml, 332 BAU/ml and 218 BAU/ml, respectively). Significant differences were found in P2, P3, and P4, but only between individuals with systemic adverse events and individuals without adverse events, while there was no statistically significant significance in P5 (**Figure 3**). In contrast, in previously infected individuals, no differences in anti-S antibody concentrations were observed between individuals who reported adverse events and those who did not. The percentage of HCP reporting adverse effects after the second dose was similar in naïve and previously infected individuals (in the naïve group: 54% no adverse events, 10% with local adverse events, 36% with systemic adverse events, in the previously infected group: 58% no adverse events, 12% with local adverse events, 30% with systemic adverse events).

## 4 Discussion

**Figure4:**
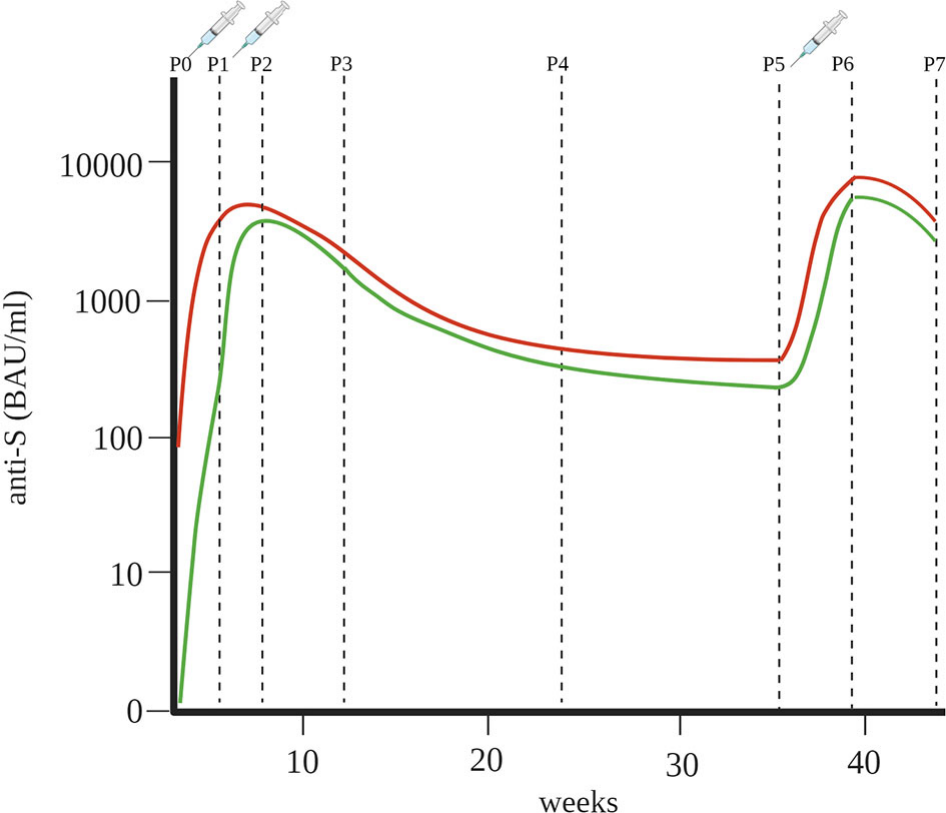
Key illustration of differences in humoral immune response between naïve individuals (green line) and previously infected individuals (red line) after the first, second and third doses of BNT162b2 (lines connect median anti-S antibody concentrations at different time points) P0 – time point before vaccination, P1 – time point three weeks after first dose, P2 – time point three weeks after second dose, P3 – time point three months after vaccination, P4 – time point six months after vaccination, P5 – time point nine months after vaccination, P6 – time point three weeks after third dose, P7 – time point three months after third dose.

In our longitudinal study we presented data about the anti-S antibody concentrations up to twelve months after vaccination with two doses of BNT162b2 as well as following the third (booster) dose in HCP (**Figure 4**).

We found that previously infected individuals had higher concentrations of anti-S antibodies after the first dose (median 3648 BAU/ml) than naïve individuals (median 253 BAU/ml). This is consistent with the fact that almost all previously infected individuals already had anti-S antibodies before vaccination (median 90 BAU/ml), so their first vaccination actually represented their second exposure to viral proteins. Therefore, similar concentrations of anti-S antibodies (median 3216 BAU/ml) were found in naïve individuals after the second dose. Appelman et al. reported that antibody concentrations increased 4.1-fold in naïve individuals and 0.97-fold in previously infected individuals after the second dose (18). In our study, the increases were 13- and 1.1-fold, respectively. Some reports indicated that previously infected individuals had higher anti-S antibody concentration after the second dose (19), while one study reported that one week after the second dose, the naïve group and previously infected group had the same median anti-S antibody concentration (20). Overall, it has already been shown that the humoral immune response to vaccination differs significantly between individuals previously infected with SARS-CoV-2 and naïve individuals (17, 19, 21–23). An important finding of this study is that the second dose did not contribute to a significant increase in anti-S antibody concentrations in previously infected individuals and that the second dose may not be essential, as suggested in other studies (17, 24, 25), or may be postponed to a later time. This also justifies vaccination of naïve individuals with two doses, as third encounter in a row does not lead to an additional increase in antibody concentrations.

Consistent with these studies, our study also showed that the concentrations of anti-S antibodies in previously infected individuals after the first dose were comparable to those who had been vaccinated twice without prior SARS-CoV-2 infection. Thus, it can be concluded that infection is equivalent to one vaccination.

In our group, there were two HCP who did not develop anti-S antibodies after vaccination with BNT162b2, most likely because of taking immunosuppressive drugs due to chronic disease. In such cases, passive immunization with monoclonal antibodies, convalescent plasma (26, 27), or antiviral agents such as Remdesivir, Paxlovid is recommended at the onset of the first symptoms of COVID-19 to prevent severe complications.

Anti-S antibody concentrations decreased after 9 months from peak concentrations (median 3216 BAU/ml in naïve individuals and median 4503 BAU/ml in previously infected individuals) to the lowest point of anti-S antibody concentrations (median 232 BAU/ml in naïve individuals and median 507 BAU/ml in previously infected individuals). An additional decrease was observed 12 months after vaccination in 22 HCP, in whom anti-S antibody concentration dropped to a median value of 229 BAU/ml in previously infected, whereas the naïve individuals had a median anti-S antibody concentration of 83 BAU/ml.

The dynamics of humoral immune response in our study are consistent with the results of certain published studies. In a clinical trial of BNT162b2, similar results were obtained on anti-S antibody concentrations, with a significant increase in naïve individuals after the first vaccination (from 1 to 913 U/ml) and after the second vaccination (from 913 to 6466 U/ml – three weeks after the second dose). The decrease from 8279 U/ml, one week after second dose (29 days after the first dose), to 2543 U/ml, 85 days after first dose, was observed in naïve individuals in a clinical study (11).

Shrotri et al. (28) showed a decrease in anti-S antibody concentration from 7506 U/ml to 3320 U/ml from 21-41 days to 70 days after the second vaccination. Favresse et al. found a significant decrease in anti-S antibody concentrations after 3 months (29), and, interestingly Israel et al. showed in their 6-month study, with 2,653 individuals included that antibodies decreased by up to 40% in each subsequent month (30). There is also evidence that the early humoral immune response may be inversely associated with antibody concentrations 90 days after vaccination (31).

Little is known about humoral immune responses after the third (booster) dose. The available data refer to patients with various diseases or specific treatments that inhibit the immune system (32, 33). However, Falsey et al. presented data from a Pfizer clinical trial data with a small number of individuals (34). The recent study from Israel showed that a third dose of BNT162b2 mRNA vaccine is effective in protecting against severe COVID-19-related outcomes, compared with only two doses given at least 5 months earlier, but did not report concentrations of antibodies after the third dose (35). We found that the third dose resulted in an even higher concentrations of anti-S antibodies compared to those measured after the second dose, which is consistent with Falsey et al. (34), and that the naïve and previously infected groups reached similar anti-S antibody concentrations. We also found that those who were less responsive after the second dose had higher concentrations of anti-S antibodies after the third dose. We can explain this phenomenon in part by the kinetics of the humoral immune response after vaccination. The antibody response increases with each vaccine booster and/or infection, but eventually a plateau is reached. Accordingly, in some individuals a plateau may have been reached after the second dose and the third dose did not result in a significant increase, whereas in others the third dose contributed significantly to the antibody response. Another confirmation of this theory is the fact that 8 participants of this study who became infected after full vaccination had anti-S antibody concentrations comparable to those already reached after the third dose. This also indicates that infection could equal one vaccine dose.

As mentioned above, in our real-world study, we found a significant decrease in humoral immune response over a 12-month period. The influence of age, sex, BMI, and previous infection with anti-S antibodies were analyzed with a mixed-model analysis. We found that previously infected individuals had higher anti-S antibody concentrations in group 1 and group 2, whereas this influence was no longer found after third dose (group 3). The reason could be the plateau of the immune system, as described above. In addition, we found that in group 1 (time points P1 and P2) and group 2 (time points P2, P3, P4, and P5) anti-S antibody concentrations were higher in individuals younger than 45 years. Similar results were found in other studies in which anti-S antibody concentrations were lower in the elderly after the second dose (13, 36–38). On the contrary, after the third dose in group 3 (time points P6 and P7), the higher anti-S antibody concentrations were found in the individuals older than 45 years (however, the oldest individuals in our group 3 had 63 years with the median age in the group of 54 years), pointing to effectiveness and relevance of third dose for elder people. However, the influence of age was lost in naïve individuals of group 3. The influence of BMI on anti-S antibody concentrations was observed only in group 3, after third dose. Levin et al. has shown that neutralizing antibodies are influenced by BMI (≥30), but after the second dose, which we did not observe. The mechanism of humoral immune response to SARS-CoV-2 in obese individuals is still unclear. Some studies have also reported lower antibody concentrations in males (28, 36, 38), but this was not the case in our study. One of the reasons for this could be the small number of men who participated in our study.

Another important finding of our study is that the high concentration of anti-S antibodies measured in individuals after they received the third dose of BNT162b2 did not protect them from SARS-CoV-2 infection, the Omicron variant. This is consistent with studies reporting that the current BNT162b2 vaccine does not effectively prevent the infection with the new variants of SARS-CoV-2 (39).

Headache, fatigue, fever, and muscle pain were the most common adverse events reported by 46% of study participants. The proportion of subjects reporting adverse events in other studies varied from 66% to 93% (13, 25). Like one other study, we have shown that the individuals with adverse events had higher concentrations of anti-S antibodies (13).

Our study exhibits several strengths. To our knowledge this is the first study investigating antibody dynamics following vaccination in real-world settings for 12-months using mixed-model analysis. Also, we examined the antibody responses separately in naïve individuals and in previously infected individuals. This has revealed that separate vaccine protocols for naïve and previously infected individuals might be feasible.

This study also has certain limitations. The first limitation is the relatively small number of participants, especially pre-vaccination samples, since the study was conducted at a single center and because of rapid vaccination implementation in our Medical Centre. However, due to urgency of providing information on antibody responses after vaccination, the results of this study are very important as they comprise new and potentially very useful data for future vaccination protocols and thus should be published timely. The second important note is that the study was conducted in an apparently healthy population of HCP and that the disease course of included previously infected individuals was rather mild. Thus we do not provide information on humoral immune response of different patient population. The third limitation of the study is that the neutralizing properties of the antibodies were not evaluated.

Our study contains several important conclusions. First, under real world conditions, we confirmed that the different vaccination schedules are appropriate for previously infected individuals, as their concentrations after the first dose were as high as in naïve individuals after the second dose. Second, we found that anti-S antibody concentrations were consistently higher in previously infected individuals than in naïve individuals at all-time points after the second dose. Third, the humoral immune response decline after the second dose and also after the third dose. Fourth, participants with lower concentrations after the second dose achieved higher concentrations after the third dose. Fifth, age had a significant effect on the humoral immune response after vaccination. Younger individuals had higher anti-S antibody concentrations after the first and second doses. Sixth, 24 HCP became infected during the 1-year observation period, five of whom became infected after the booster dose. Seventh, high anti-S antibody concentrations did not effectively prevent the infection with Omicron variant, and eight, individuals with systemic adverse events achieved higher concentrations after the second dose than those with local or no adverse events.

Our study provides new data on the response to vaccination after three doses of BNT162b2 and reports the decline in anti-S antibodies in naïve and previously infected individuals, comprising real data from a 12-month observation period using mixed-model analysis. Because of the many factors that influence humoral response and the intervariability between individuals, such studies are important to confirm previously reported data and to substantiate the new findings. Furthermore, we show that the high concentrations of anti-S antibodies were not protective against new variants of SARS-CoV-2, indicating the need to optimize vaccines for the different variants.

## Data Availability Statement

Raw data supporting the conclusions of this article will be made available by the authors, without undue reservation.

## Ethics Statement

The studies involving human participants were reviewed and approved by Slovenian National Medical Ethics Committee (#0120-422/2020/6). The participants provided their written informed consent to participate in this study.

## Author Contributions

MO, ŽR and SČ participated in research design. MO participated in data curation and sample acquisition. MO, PŽ, EP and KL participated in data analysis. MO, PŽ, KL and SČ participated in the writing of the paper. EP, SS-S and ŽR participated in the review and editing of the paper. SS-S, ŽR and SČ participated in funding acquisition and project administration. All authors contributed to the article and approved the submitted version.

## Funding

The study was supported by the University Medical Centre Ljubljana with a tertiary project and by the Slovenian Research Agency ARRS with the National Research Program #P3-0314.

## Conflict of Interest

The authors declare that the research was conducted in the absence of any commercial or financial relationships that could be construed as a potential conflict of interest.

## Publisher’s Note

All claims expressed in this article are solely those of the authors and do not necessarily represent those of their affiliated organizations, or those of the publisher, the editors and the reviewers. Any product that may be evaluated in this article, or claim that may be made by its manufacturer, is not guaranteed or endorsed by the publisher.

## Supplementary Material

Supplementary Table 1 Mixed-model analysis of variables (age, sex, body mass index (BMI), previous infection and time) and anti-S antibody concentrations in group 1, group 2 and group 3, presented first for the entire group and then for the naïve individuals only.
