# Supplementary material for: Differences in SARS-CoV-2-Specific Antibody Responses After the First, Second, and Third Doses of BNT162b2 in Naïve and Previously Infected Individuals: A 1-Year Observational Study in Healthcare Professionals
Source: Front Immunol. 2022 May 27;13:876533. doi: 10.3389/fimmu.2022.876533 (PMC9196038; doi:10.3389/fimmu.2022.876533)
Supplement: Supplementary Table 1 — Mixed-model analysis of variables (age, sex, body mass index (BMI), previous infection and time) and anti-S antibody concentrations in group 1, group 2 and group 3, presented first for the entire group and then for the naïve individuals only. [file Table_1.pdf]

Supplementary Table 1 | Mixed-model analysis of variables (age, sex, body mass index (BMI), previous infection and time) and anti-S antibody concentrations in group 1, group 2 and group 3, presented first for the entire group and then for the naïve individuals only.

		**Group 1**													
		**all**							**naïve individuals**						
		**Estimate**	**Std. Error**	**df**	**t**	**Sig.**	**95% Confidence Interval (lower bound - upper bound)**		**Estimate**	**Std. Error**	**df**	**t**	**Sig.**	**95% Confidence Interval (lower bound - upper bound)**	
**age**	**<45 years**	1595.02	793.28	34.81	2.01	0.052	-15.73	3205.77	1059.00	474.17	23.70	2.23	**0.035**	**79.72**	**2038.28**
	**>45 years**	0.00	0.00						0.00	0.00					
**sex**	**female**	1364.78	1039.79	34.79	1.31	0.198	-746.57	3476.12	-48.65	616.67	23.64	-0.08	0.938	-1322.42	1225.12
	**male**	0.00	0.00						0.00	0.00					
**BMI**	**<25**	428.97	832.97	34.76	0.51	0.610	-1262.47	2120.40	82.97	509.14	23.69	0.16	0.872	-968.56	1134.51
	**>25**	0.00	0.00						0.00	0.00					
**previous infection**	**no**	-3941.66	889.38	34.81	-4.43	**0.000**	**-5747.56**	**-2135.77**							
	**yes**	0.00	0.00												
**time**		83.22	24.78	41.70	3.36	**0.002**	**33.20**	**133.23**	126.70	15.79	27.91	8.02	**0.000**	**94.35**	**159.06**
		**Group 2**													
**age**	**<45 years**	398.58	167.66	244.72	2.38	**0.018**	**68.35**	**728.82**	341.93	166.97	211.56	2.05	**0.042**	**12.80**	**671.06**
	**>45 years**	0.00	0.00						0.00	0.00					
**sex**	**female**	356.38	206.71	249.51	1.72	0.086	-50.73	763.50	161.93	206.01	215.00	0.79	0.433	-244.13	567.99
	**male**	0.00	0.00						0.00	0.00					
**BMI**	**<25**	-213.11	164.80	243.38	-1.29	0.197	-537.73	111.51	-147.16	165.39	210.14	-0.89	0.375	-473.20	178.87
	**>25**	0.00	0.00						0.00	0.00					
**previous infection**	**no**	-938.32	242.70	264.84	-3.87	**0.000**	**-1416.18**	**-460.46**							
	**yes**	0.00	0.00												
**time**		-15.58	0.72	979.01	-21.62	**0.000**	**-16.997**	**-14.168**	-14.68	0.72	857.66	-20.48	**0.000**	**-16.09**	**-13.27**
		**Group 3**													
**age**	**<45 years**	-1205.89	528.46	99.76	-2.28	**0.025**	**-2254.38**	**-157.40**	-1069.34	567.37	91.24	-1.88	0.063	-2196.32	57.64
	**>45 years**	0.00	0.00						0.00	0.00					
**sex**	**female**	-25.49	664.77	92.76	-0.04	0.969	-1345.64	1294.65	-55.99	706.66	83.79	-0.08	0.937	-1461.31	1349.34
	**male**	0.00	0.00						0.00	0.00					
**BMI**	**<25**	-1717.47	537.22	105.71	-3.20	**0.002**	**-2782.58**	**-652.35**	-1637.60	574.98	94.46	-2.85	**0.005**	-2779.16	-496.04
	**>25**	0.00	0.00						0.00	0.00					
**previous infection**	**no**	-522.62	877.14	97.73	-0.60	0.553	-2263.33	1218.09							
	**yes**	0.00	0.00												
**time**		-8.40	3.52	148.30	-2.39	**0.018**	**-15.36**	**-1.45**	-7.25	3.65	132.60	-1.99	**0.049**	**-14.47**	**-0.04**
